# StitchR-based dual-AAV delivery strategies enable efficient transduction of defined neural cell populations

**DOI:** 10.1186/s13041-026-01331-0

**Published:** 2026-07-24

**Authors:** Nengsong Luo, Yunling Gao, Zengpeng Han, Peng Su, Fuqiang Xu, Kunzhang Lin

**Affiliations:** 1 Shenzhen Key Laboratory of Viral Vectors for Biomedicine, Shenzhen-Hong Kong Institute of Brain Science, Shenzhen Institutes of Advanced Technology, Chinese Academy of Sciences, Shenzhen, 518055 People’s Republic of China; 2 Wuhan National Laboratory for Optoelectronics, Huazhong University of Science and Technology, Wuhan, 430074 People’s Republic of China; 3 Key Laboratory of Magnetic Resonance in Biological Systems, State Key Laboratory of Magnetic Resonance and Atomic and Molecular Physics, National Center for Magnetic Resonance in Wuhan, Innovation Academy for Precision Measurement Science and Technology, Chinese Academy of Sciences, Wuhan, 430071 People’s Republic of China; 4 Institute of Neuroscience and Brain Diseases, Xiangyang Central Hospital, Affiliated Hospital of Hubei University of Arts and Science, Xiangyang, 441106 People’s Republic of China; 5 Key Laboratory of Quality Control Technology for Virus-Based Therapeutics, Guangdong Provincial Medical Products Administration, NMPA Key Laboratory for Research and Evaluation of Viral Vector Technology in Cell and Gene Therapy Medicinal Products, the Brain Cognition and Brain Disease Institute, Shenzhen Institutes of Advanced Technology, Chinese Academy of Sciences, Shenzhen, 518055 People’s Republic of China; 6 Brain Case Biotechnology Co., Ltd., Shenzhen, 518107 People’s Republic of China

**Keywords:** StitchR, Neuron, Astrocyte, Microglia, AAV11, AAVhu.32-PLUS, Dual AAV, Blood–brain barrier

## Abstract

Dual adeno-associated virus (AAV) vector reconstitution has become an indispensable technique for delivering large genes in neuroscience and gene therapy. A novel technology termed StitchR addresses the challenge of low efficiency in dual-AAV reconstruction, yet related tools enabling targeted gene delivery to specific neural cell populations remain insufficiently developed. Herein, we developed four StitchR-based dual-viral reconstitution strategies by combining high-efficiency AAV serotypes with neuron- or glia-specific promoters. Specifically, the AAV11-hSyn-driven StitchR strategy achieved specific transduction of local neurons and their upstream projection neurons (≥ 94.44% specificity); the AAV11-GfaABC1D-driven strategy realized astrocyte-specific transduction (approximately 98.25% specificity); and the AAV11-mIBA1-driven strategy enabled microglia-specific transduction (100% specificity). Furthermore, by incorporating the blood–brain barrier (BBB)-penetrating AAVhu.32-PLUS into the hSyn-driven system, we established a modified strategy that allows brain-wide neuronal transduction. Finally, we confirmed that the StitchR-based dual-viral reconstitution strategy can achieve efficient functional reconstitution of large transgenes in neural cells. Collectively, these dual-AAV delivery strategies expand the valuable AAV toolbox and offer crucial technical support for targeted delivery of large transgenes to defined neural cell populations, holding great significance for basic neuroscience research and potential therapeutic applications.

**Supplementary Information:**

The online version contains supplementary material available at https://doi.org/10.1186/s13041-026-01331-0.

## Introduction

The brain is the most complex organ in the human body and profoundly influences cognitive functions, behaviour, and emotion [1]. Neuroscience, by investigating the brain’s structure, function, and interactions among neurons, has revealed key insights into memory, learning, perception, decision-making, social interactions, and psychiatric disorders. These findings have had far-reaching impacts across medicine, psychology, artificial intelligence, and education, and they provide essential theoretical support for the diagnosis and treatment of neurological diseases such as Parkinson’s disease and Alzheimer’s disease [2, 3]. With ongoing advances in science and technology, neuroscience will further deepen our understanding of the brain and furnish a robust theoretical foundation and practical guidance for treating neurological disorders, improving quality of life, and driving technological progress.

Deciphering the neural circuits related to development, behaviour, and learning and memory is one of the core objectives of neuroscience research. Through genetic manipulation, researchers can probe the mechanisms that encode neural behaviour, elucidate the functions of specific neuronal populations and brain regions, and determine the roles of molecular components within neural circuits. CRISPR–Cas9 technology provides a powerful tool for gene editing and functional studies [4], and gene therapy—delivering exogenous genes as therapeutic agents—has been successfully applied to hereditary disorders such as cystic fibrosis [5] and muscular dystrophy [6]. Achieving these aims depends critically on efficiently delivering target genes into specific tissues or cell types, which in turn relies on the availability of suitable gene-delivery vectors. Viral vectors—particularly adeno-associated viruses (AAVs)—have emerged as one of the preferred gene delivery vehicles due to their low toxicity, low immunogenicity, capacity for long-term transgene expression, and broad tissue tropism [7], and have been extensively utilized for treating rare diseases affecting the eye, liver, brain, muscle, heart, and other tissues [8]. To date, nine gene therapy products have obtained regulatory approval—further validating the safety and efficacy of AAVs as gene delivery vectors [9]. Although AAVs are widely used in clinical trials and basic research, it is well known that their packaging capacity is limited. Because of their small capsid size (≈250 Å in diameter), they can reliably package and produce genomes of only up to ~ 4.7 kb, including regulatory elements such as promoters and viral recognition sequences. This constraint impedes the delivery of large sequences and thus poses a challenge for gene-replacement (gene-supplementation) therapies. Many disease-causing genes that are potential targets for such therapies exceed this packaging limit. Moreover, the restricted packaging capacity extends to most CRISPR tools developed for applications in genome and epigenome editing. To address these challenges, dual AAV vectors utilizing StitchR-mediated reconstitution have been developed for delivering large genes (e.g., full-length human dysferlin) in the treatment of Duchenne muscular dystrophy [10], yet corresponding tools for targeted gene delivery to specific neural cell populations remain undeveloped.

Here, we established and optimized an efficient AAV-based gene delivery platform that integrates the StitchR-mediated gene reconstitution strategy, cell-type-specific promoters and high-performance AAV capsids, aiming to evaluate the feasibility of delivering genetic payloads and enabling effective expression in distinct brain cell types. After in vitro functional validation, this system was further systematically characterized in vivo. For neuron-targeted delivery, we employed the natural serotype AAV11 [11], constructed a neuron-specific hSyn-driven StitchR-based dual AAV reconstitution strategy, which enabled robust transduction of local neurons and retrograde labeling of upstream projection neurons. For astrocyte-targeted delivery, we developed GfaABC1D-driven StitchR-based dual AAV11 vectors, which upon co-injection into the striatum yielded strong, highly specific EGFP expression in astrocytes. For microglia-targeted delivery, we engineered AAV11 to incorporate a microglia-specific promoter and a neuron-detargeting element [12]. Co-injection of the mIBA1-driven StitchR-based dual AAV11 vectors achieved efficient, exclusive EGFP expression in microglia. For brain-wide neuronal delivery, we utilized the engineered BBB-penetrant capsid AAVhu.32-PLUS [13]. Systemic co-injection of hSyn-driven StitchR-based dual AAVhu.32-PLUS vectors via the tail vein achieved widespread EGFP expression in neurons across multiple brain regions.

In summary, by rationally combining cell-type-specific expression cassettes, customized AAV capsids, and optimized delivery routes, this platform facilitates precise delivery of target genes to neurons, astrocytes, and microglia, providing a scalable and versatile framework to underpin large-gene applications in neuroscience research and gene therapy.

## Results

### Construction and validation of the dual-expression cassette strategy

**Fig. 1:**
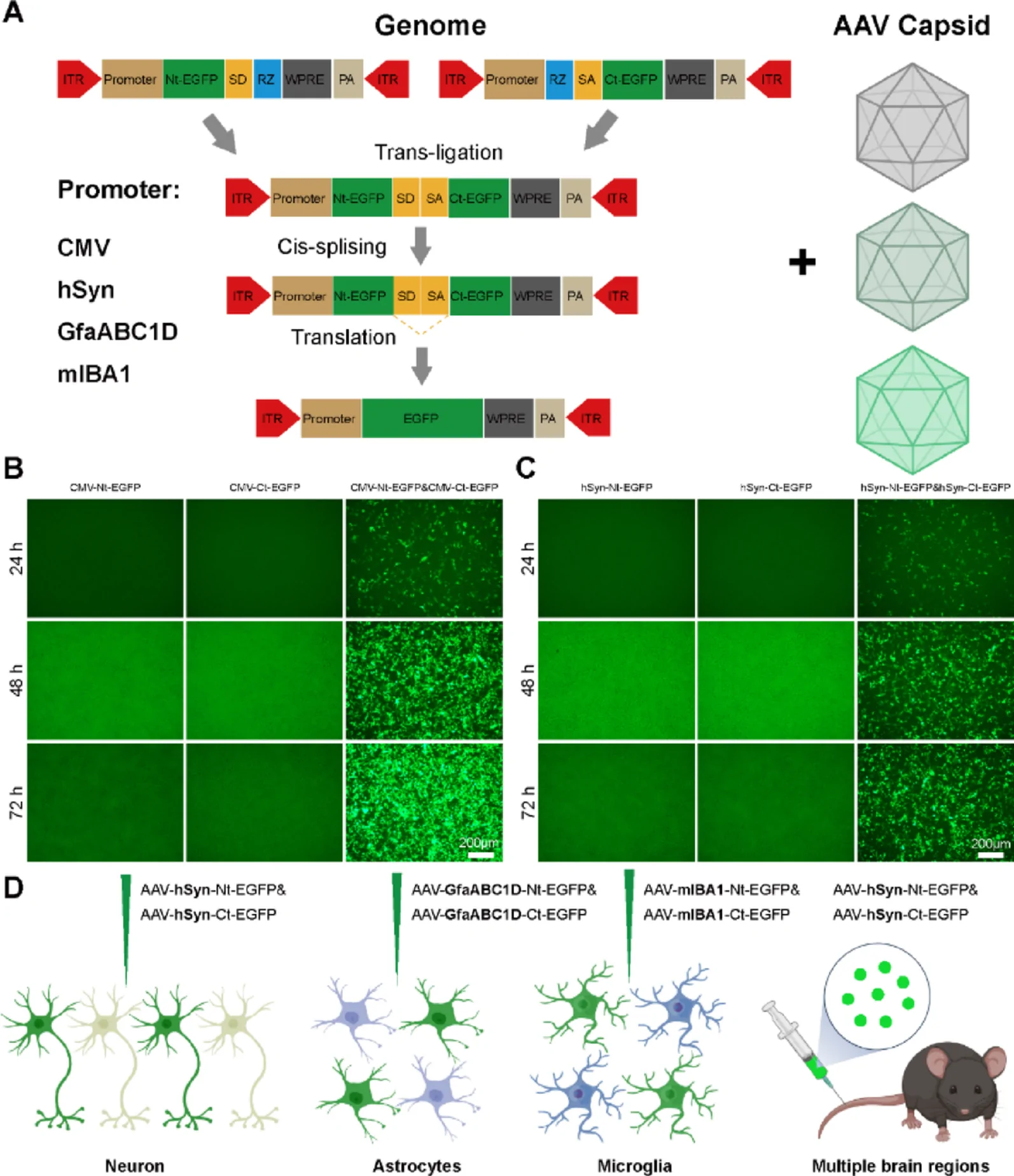
Construction and validation of the StitchR-based dual AAV vector system. **A** Design of a dual-AAV system based on StitchR RNA trans-ligation. The EGFP open reading frame (ORF), containing an intron with splice donor (SD) and splice acceptor (SA) sites, was split into non-overlapping N-terminal (Nt) and C-terminal (Ct) fragments. Small catalytic ribozymes (Rz) were placed at the 3′ end of the Nt-GFP fragment and the 5′ end of the Ct-GFP fragment to generate precise RNA termini and enable scarless trans-ligation. Promoters can be chosen from CMV (ubiquitous), hSyn (neuron-specific), GfaABC1D (astrocyte-specific) or mIBA1 (microglia-specific), and capsids can be selected from libraries of natural or engineered AAVs with differing tropisms. The combination of genome design and capsid determines efficient, cell-type-specific transduction and RNA reconstitution in target neural cells. **B** In vitro functional validation of StitchR-based dual AAV plasmids driven by the CMV promoter. Transfection with either pAAV-CMV-Nt-EGFP or pAAV-CMV-Ct-EGFP alone produced no GFP signals; GFP fluorescence signals were only observed when both plasmids were co-transfected, indicating correct intracellular reconstitution of functional EGFP. **C** In vitro functional validation of StitchR-based dual AAV plasmids driven by the hSyn promoter. Neither pAAV-hSyn-Nt-EGFP nor pAAV-hSyn-Ct-EGFP alone yielded GFP expression; GFP was detected only upon co-transfection, confirming successful GFP reconstitution. **D** Schematic of in vivo dual-vector delivery strategies using different promoters. In situ (local) injection is used to target neurons in the injection locus and upstream regions, as well as astrocytes and microglia at the injection site; systemic tail-vein administration with BBB-penetrant capsids is used to achieve widespread neuronal delivery across the brain

Mammalian cells possess an intrinsic ability to catalyze the trans-ligation of separately cleaved mRNA fragments: ribozymes act as “molecular scissors” to generate distinct 5′-OH and 2′,3′-cyclic phosphate (2′,3′-cP) termini that are subsequently recognized and joined by cellular RNA ligases such as RtcB [10]. StitchR expands the RNA toolbox and provides a programmable approach for generating chimeric or full-length proteins for numerous research and therapeutic applications. To validate whether StitchR can be coupled with AAV vectors for targeted gene delivery to specific neural cell populations in the mouse brain, we established StitchR-based dual-viral reconstitution strategies. Selected promoters included the ubiquitous CMV promoter for in vitro testing, and a panel of cell-type-specific promoters: the neuron-specific hSyn, astrocyte-specific GfaABC1D, and microglia-specific mIBA1 (Fig. 1A).

To assess the feasibility of delivering transgenes to the brain’s most commonly studied cell types (neurons, astrocytes, and microglia), AAV capsids can be chosen from natural serotypes such as AAV11, which we previously showed efficiently transduces neurons in situ and retrogradely labels projecting neurons in upstream regions, and when combined with cell-type promoters can specifically transduce astrocytes and microglia. In addition, AAVhu.32-PLUS may be used to explore gene delivery across the BBB.

To evaluate the functionality of the core plasmids, CMV- and hSyn-driven constructs were first tested in HEK-293 T cells in vitro. Transfection of Nt-EGFP or Ct-EGFP alone yielded no detectable EGFP signals (Fig. 1B, C); only co-transfection of the two constructs restored EGFP signals (Fig. 1B, C), providing initial evidence that the core plasmids function as designed. Recombinant AAVs were packaged via a triple-plasmid transient transfection protocol, and their transduction properties were subsequently validated in male C57BL/6 mice; the in vivo validation workflow is shown in Fig. 1D.

### A hSyn-driven StitchR-based dual AAV reconstitution strategy for specifically transducing local neurons and their upstream projecting neurons

**Fig. 2:**
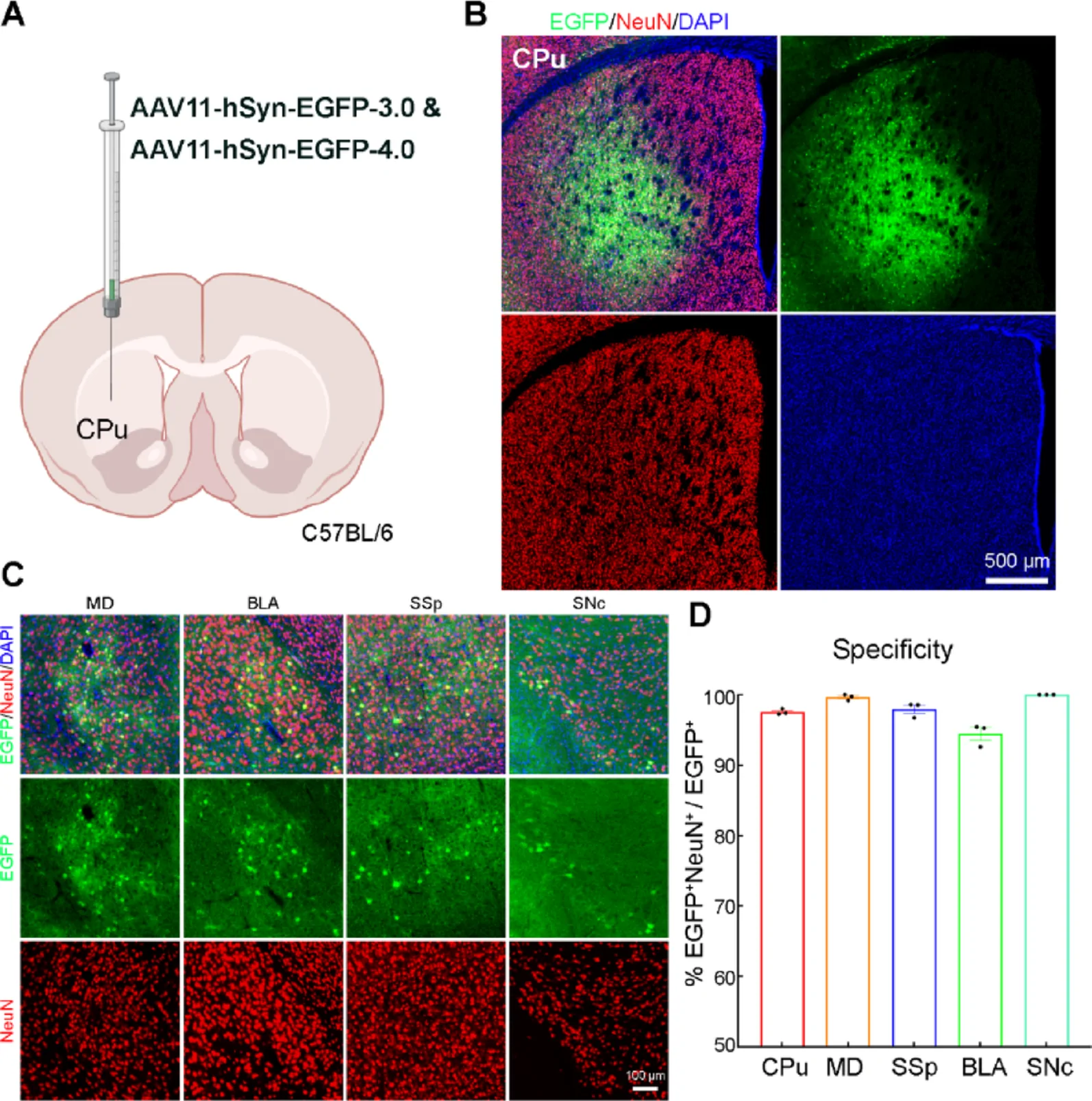
In vivo validation of hSyn-driven StitchR-based dual AAV vector transduction. **A** Schematic diagram of virus injection. A dual-vector system driven by the neuron-specific hSyn promoter was assembled with AAV11 capsids, and both vectors (each 2 × 10.^9^ VG) were locally injected into the CPu region of C57BL/6 mice (*n* = 4) to evaluate in vivo reconstitution and expression efficiency. For simplicity of description, AAV11-hSyn-Nt-EGFP-SD-RZ-WPRE-PA is hereafter referred to as AAV11-hSyn-EGFP-3.0, and AAV11-hSyn-RZ-SA-Ct-EGFP-WPRE-PA is referred to as AAV11-hSyn-EGFP-4.0 throughout this study. **B** Imaging and cellular identification at the injection site. Robust EGFP fluorescence signals were observed in the CPu region. Immunofluorescent staining for NeuN in the same region showed that most EGFP-positive cells were neurons, confirming cell-type specificity of transduction. Scale bar = 500 μm. **C** EGFP-positive neurons were also observed in several nuclei projecting to the CPu, including the mediodorsal thalamus (MD), the basolateral amygdala (BLA), the primary somatosensory cortex (SSp), and the substantia nigra pars compacta (SNc), indicating efficient transgene expression and labeling across the connected projection circuits. Scale bar = 100 μm. **D** Quantitative analysis of the specificity of hSyn-driven StitchR-based dual AAV11 vectors in transducing neurons in the injection site and its upstream brain regions (mean ± SEM; *n* = 3)

Many central nervous system disorders require the targeted delivery of large transgenes to neurons. One prominent example is Dravet syndrome (DS), a severe, treatment-refractory epileptic encephalopathy most often caused by loss-of-function mutations in SCN1A, which encodes the voltage-gated sodium channel NaV1.1 and leads to impaired inhibitory interneuron function [14]. Numerous other neurological diseases similarly demand delivery of oversized genes to neuronal populations [15, 16]. Therefore, achieving efficient and neuron-specific delivery of large transgenes is critical for addressing these disorders. Here, we investigated whether the hSyn-driven StitchR-based dual AAV strategy can mediate transgene reconstitution in neurons. AAV11-hSyn-Nt-EGFP-SD-RZ-WPRE-pA and AAV11-hSyn-RZ-SA-Ct-EGFP-WPRE-pA were mixed in equal amounts and injected into the caudate–putamen (CPu) of mice (Fig. 2A). Three weeks after injection, animals were perfused and brain tissue was collected for fluorescence imaging. Strong GFP fluorescence signals were observed at the injection site, and immunofluorescence labeling confirmed that the majority of labeled cells were neurons (Fig. 2B). In addition, GFP signals were detected in the upstream CPu-projecting regions including the mediodorsal thalamic nucleus (MD), the primary somatosensory area (SSp), the basolateral amygdala (BLA), and the substantia nigra pars compacta (SNc); immunostaining indicated that the majority of labeled cells in these areas were neurons (Fig. 2C). Further quantitative analyses confirmed that this strategy confers remarkably high neuronal transduction specificity in the Cpu (approximately 97.56% specificity) and its upstream brain regions including the MD (approximately 99.45% specificity), the SSp (approximately 97.96% specificity), the BLA (approximately 94.44% specificity), and the SNc (100% specificity) (Fig. 2D). In summary, we demonstrate that the hSyn-driven StitchR-based dual AAV strategy can effectively and specifically express transgenes in neurons.

### A GfaABC1D-driven StitchR-based dual AAV reconstitution strategy for specifically transducing astrocytes

**Fig. 3:**
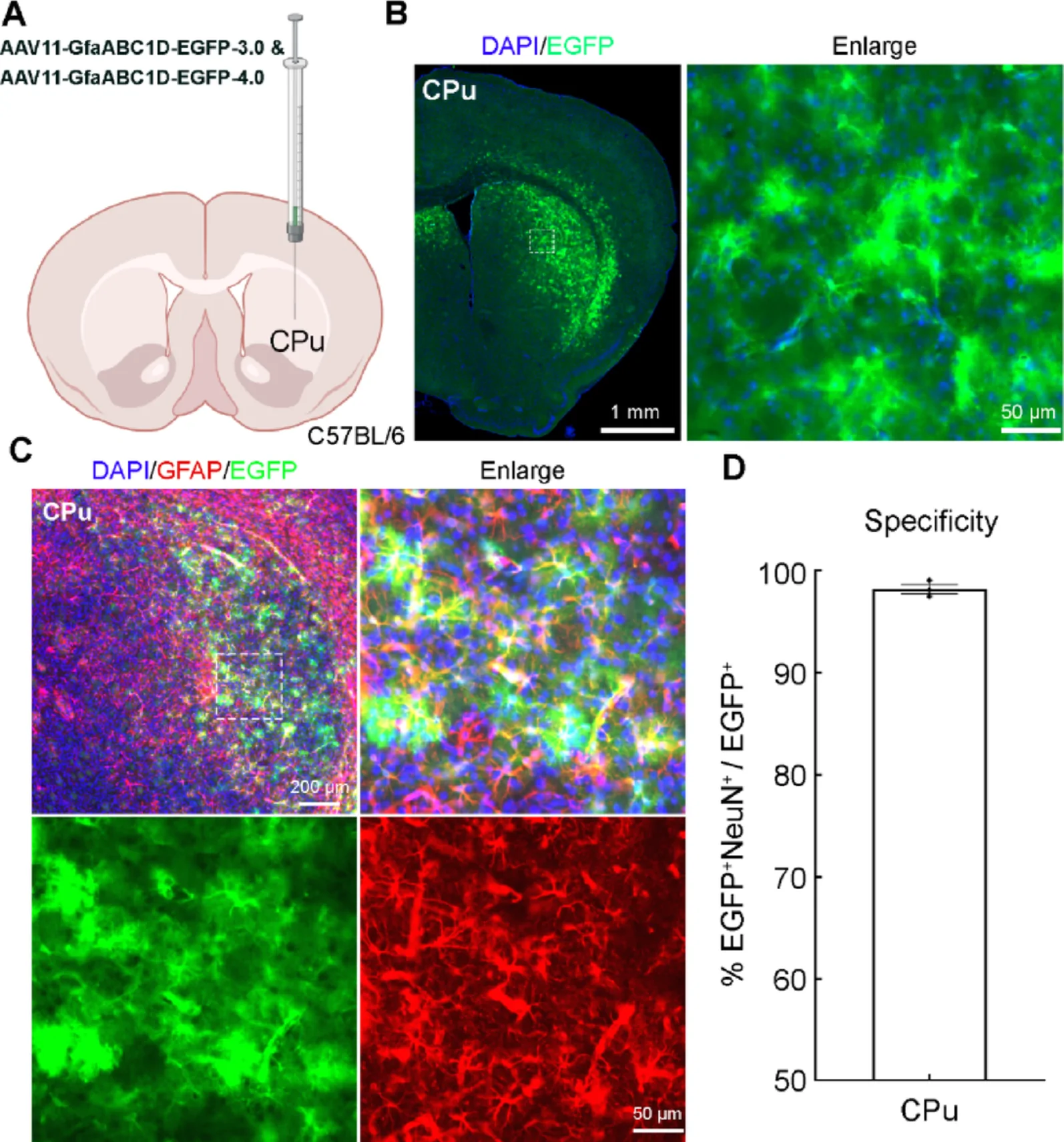
Validation of GfaABC1D-driven StitchR-based dual AAV transduction in vivo. **A** Schematic diagram of virus injection. A dual-vector system driven by the astrocyte-specific GfaABC1D promoter was assembled using AAV11 capsids. Both vectors (each 2 × 10.^9^ VG) were locally injected into the CPu region of C57BL/6 mice (*n* = 4) to evaluate in vivo reconstitution and expression efficiency. For brevity, AAV11-GfaABC1D-EGFP-3.0 denotes AAV11-GfaABC1D-Nt-EGFP-SD-RZ-WPRE-PA, and AAV11-GfaABC1D-EGFP-4.0 denotes AAV11-GfaABC1D-RZ-SA-Ct-EGFP-WPRE-PA. **B** Fluorescence imaging at the injection site. Robust EGFP fluorescence signals were observed in the CPu area. Scale bar = 1 mm (left 1 panel), 50 μm (right 1 panel). **C** Cell-type identification of labeled cells. Immunofluorescent staining for GFAP in the same region showed that most EGFP-positive cells were GFAP-positive astrocytes, confirming astrocyte-specific transduction. Scale bar = 200 μm (left 1 panel), 50 μm (right 1 panel and bottom 2 panels). **D** Quantitative analysis of the specificity of GfaABC1D-driven StitchR-based dual AAV11 vectors in transducing astrocytes (mean ± SEM; *n* = 3)

Astrocytes, the most abundant glial cells in the brain, are crucial for maintaining overall brain health. Many treatments for central nervous system disorders and numerous neuroscience studies require the targeted delivery of large transgenes to astrocytes [17, 18]; successful, astrocyte-specific delivery of large constructs is therefore a critical step toward addressing these needs. Here, we evaluated whether the GfaABC1D-driven StitchR-based dual AAV strategy can mediate transgene reconstitution in astrocytes. AAV11-GfaABC1D-Nt-EGFP-SD-RZ-WPRE-pA and AAV11-GfaABC1D-RZ-SA-Ct-EGFP-WPRE-pA were mixed in equal amounts and injected into the CPu of mice (Fig. 3A). Three weeks after injection, strong GFP fluorescence signals were observed at the injection site (Fig. 3B), and immunofluorescence staining confirmed that the majority of labeled cells were astrocytes (Fig. 3C). Further quantitative analyses showed that this strategy achieves exceptionally high astrocyte transduction specificity (approximately 98.25%) at the injection site (Fig. 3D). In summary, we show that the GfaABC1D-driven StitchR-based dual AAV strategy can effectively and specifically deliver and reconstitute transgenes in astrocytes.

### A mIBA1-driven StitchR-based dual AAV reconstitution strategy for specifically transducing microglia

**Fig. 4:**
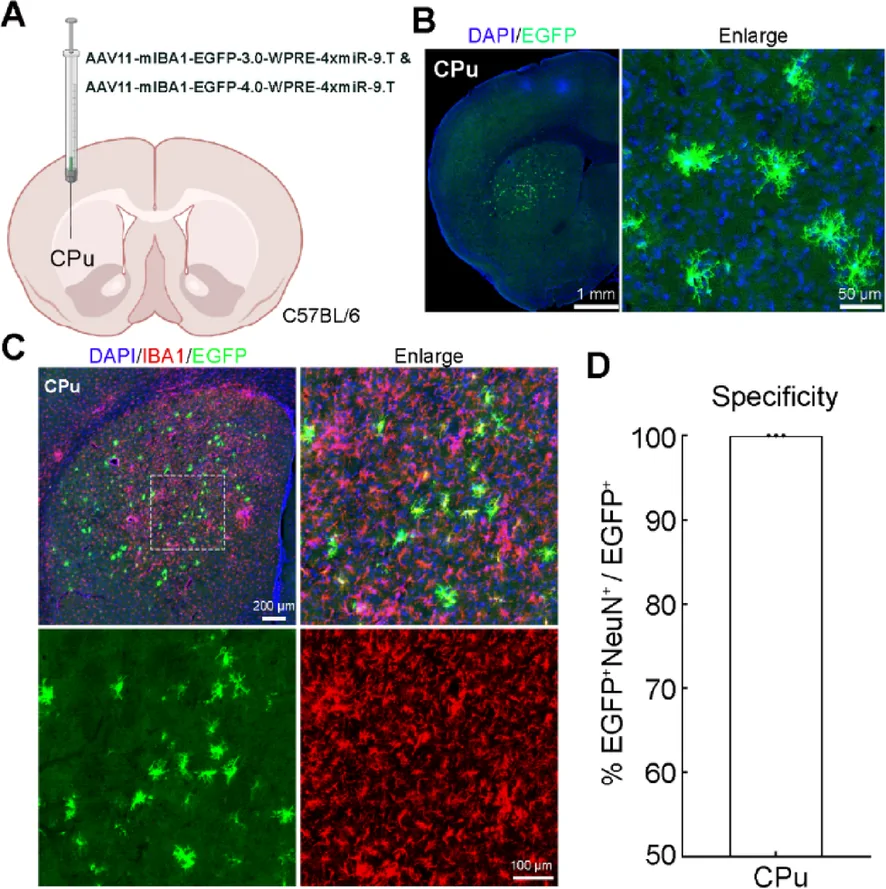
Validation of mIBA1-driven StitchR-based dual AAV transduction in vivo. **A** Schematic diagram of virus injection. A dual-vector system driven by the microglia-specific mIBA1 promoter was assembled using AAV11 capsids. Both vectors (each 2 × 10.^9^ VG) were locally injected into the CPu region of C57BL/6 mice (*n* = 4) to evaluate in vivo reconstitution and expression efficiency. For brevity, AAV11-mIBA1-EGFP-3.0 denotes AAV11-mIBA1-Nt-EGFP-SD-RZ-WPRE-4 × miR-9.T-PA, and AAV11-mIBA1-EGFP-4.0 denotes AAV11-mIBA1-RZ-SA-Ct-EGFP-WPRE-4 × miR-9.T-PA. **B** Fluorescence imaging at the injection site. Robust EGFP fluorescence signals were observed in the CPu injection site. Scale bar = 1 mm (left 1 panel), 50 μm (right 1 panel). **C** Cell-type identification of labeled cells. Immunofluorescent staining for IBA1 in the same region showed that all EGFP-positive cells were IBA1-positive, confirming microglia-specific transduction. Scale bar = 200 μm (left 1 panel), 100 μm (right 1 panel and bottom 2 panels). **D** Quantitative analysis of the specificity of mIBA1-driven StitchR-based dual AAV11 vectors in transducing microglia (mean ± SEM; *n* = 3)

Microglia, the brain’s resident immune cells, are essential for maintaining neural health [11, 19–21]. Many therapies for central nervous system disorders and numerous neuroscience studies require targeted delivery of large transgenes to microglia; successful, microglia-specific delivery of oversized constructs is therefore a critical step. Here, we evaluated whether the mIBA1-driven StitchR-based dual AAV strategy can mediate transgene reconstitution in microglia. AAV11-mIBA1-Nt-EGFP-SD-RZ-WPRE-4 × miR-9.T-pA and AAV11-mIBA1-RZ-SA-Ct-EGFP-WPRE-4 × miR-9.T-pA were mixed in equal amounts and injected into the CPu of mice (Fig. 4A). Three weeks after injection, animals were perfused and brain tissue collected for fluorescence imaging. Strong GFP fluorescence signals were observed at the injection site (Fig. 4B), and immunofluorescence staining identified the majority of labeled cells as microglia (Fig. 4C). Further quantitative analyses validated that this strategy yields outstandingly high microglia transduction specificity (100%) at the injection site (Fig. 4D). In summary, we demonstrate that the mIBA1-driven StitchR-based dual AAV strategy can effectively and specifically deliver and reconstitute transgenes in microglia.

### A StitchR-based blood–brain barrier-penetrating dual AAV reconstitution strategy for transducing brain-wide neurons

**Fig. 5:**
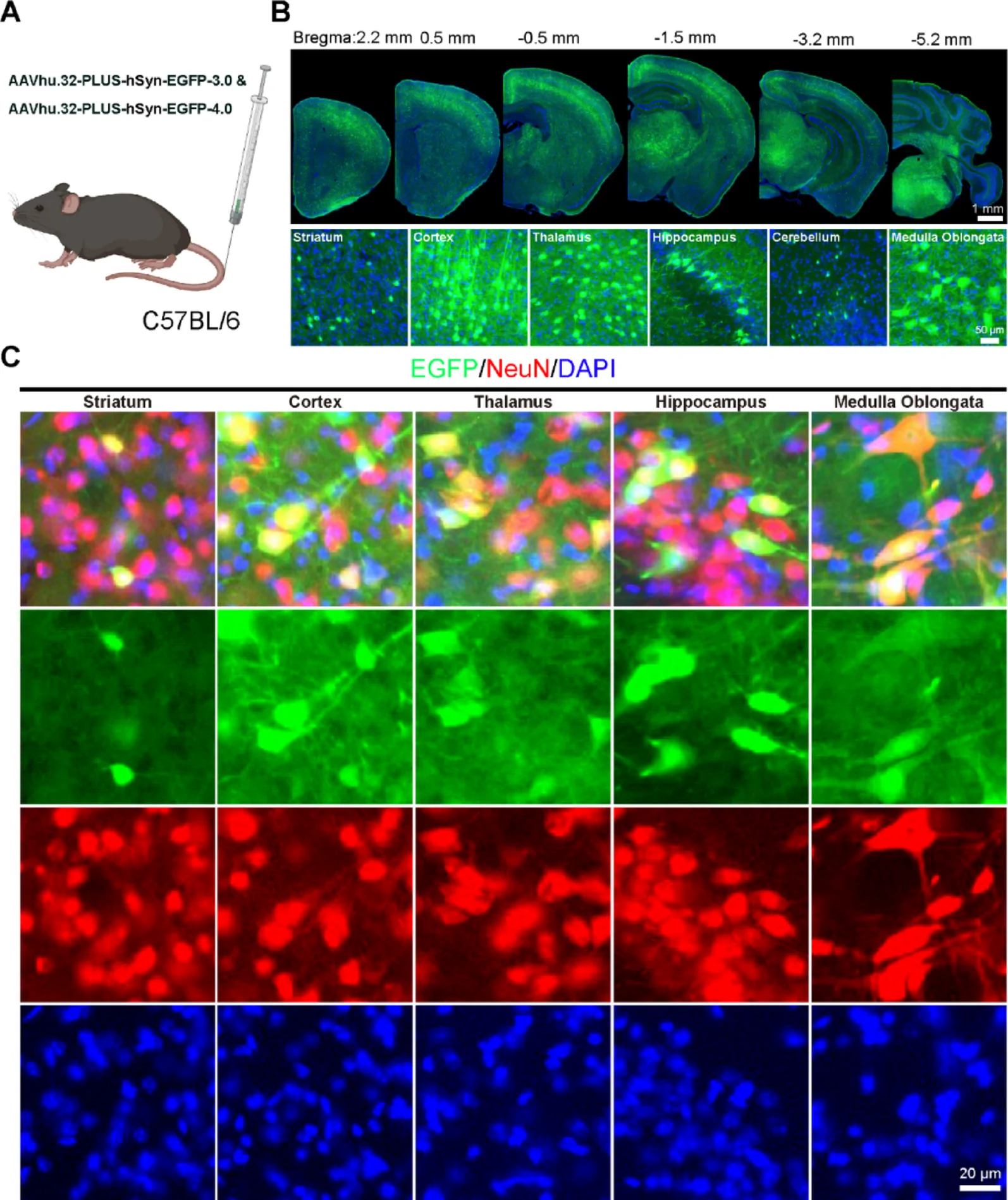
Validation of hSyn-driven StitchR-based dual AAV transduction in the whole-brain. **A** Schematic diagram of virus injection. A hSyn promoter–driven dual-vector system packaged in AAVhu.32-PLUS capsids was prepared; the two vectors (each 2 × 10^9^ VG) were mixed and co-administered via tail-vein injection into C57BL/6 mice (*n* = 4) to evaluate genome reconstitution and transgene expression in the whole-brain. For brevity, AAVhu.32-PLUS-hSyn-EGFP-3.0 denotes AAVhu.32-PLUS-hSyn-Nt-EGFP-SD-RZ-WPRE-PA, and AAVhu.32-PLUS-hSyn-EGFP-4.0 denotes AAVhu.32-PLUS-hSyn-RZ-SA-Ct-EGFP-WPRE-PA. **B** Fluorescence imaging of whole-brain sections. Robust EGFP signals were observed across multiple brain regions, including the striatum, cortex, hippocampus, thalamus, cerebellum and medulla. Scale bar = 1 mm (top 1 panel), 50 μm (bottom 6 panels). **C** Cell-type identification of labeled cells. Immunofluorescent staining for NeuN showed that all EGFP-positive cells were neurons. Scale bar = 20 μm

Many neurodegenerative diseases involve the progressive degeneration of neural networks distributed across multiple brain regions. Common disorders such as lysosomal storage diseases, Alzheimer’s disease, and Parkinson’s disease exhibit characteristic brain-wide pathological alterations [22–25]. In addition, there are numerous conditions that require delivery of large-fragment gene constructs to neurons throughout the brain for therapeutic intervention, for example, to treat virus infections [26] and similar disorders. Successful, neuron-specific delivery of large genetic payloads across the whole brain is therefore a critical prerequisite for addressing these problems. Here, we established a hSyn-driven StitchR-based blood–brain barrier-penetrating dual AAV strategy, and investigated whether it can mediate expression of transgenes at brain-wide neurons. We mixed AAVhu.32-PLUS-hSyn-Nt-EGFP-SD-RZ-WPRE-pA and AAVhu.32-PLUS-hSyn-Nt-EGFP-SD-RZ-WPRE-pA at defined doses and administered them to C57BL/6 mice by tail-vein injection (Fig. 5A). Three weeks after injection, strong green fluorescence signals were observed across multiple brain regions, including the cortex, the striatum, the hippocampus, the thalamus and the medulla (Fig. 5B). Immunofluorescence staining identified the labeled cells primarily as neurons (Fig. 5C).

In summary, we demonstrate that the hSyn-driven StitchR-based blood–brain barrier-penetrating dual AAV vector system can efficiently deliver genes to neurons throughout the brain.

### Combination of AAV and StitchR strategy for large gene delivery in neural cells

**Fig. 6:**
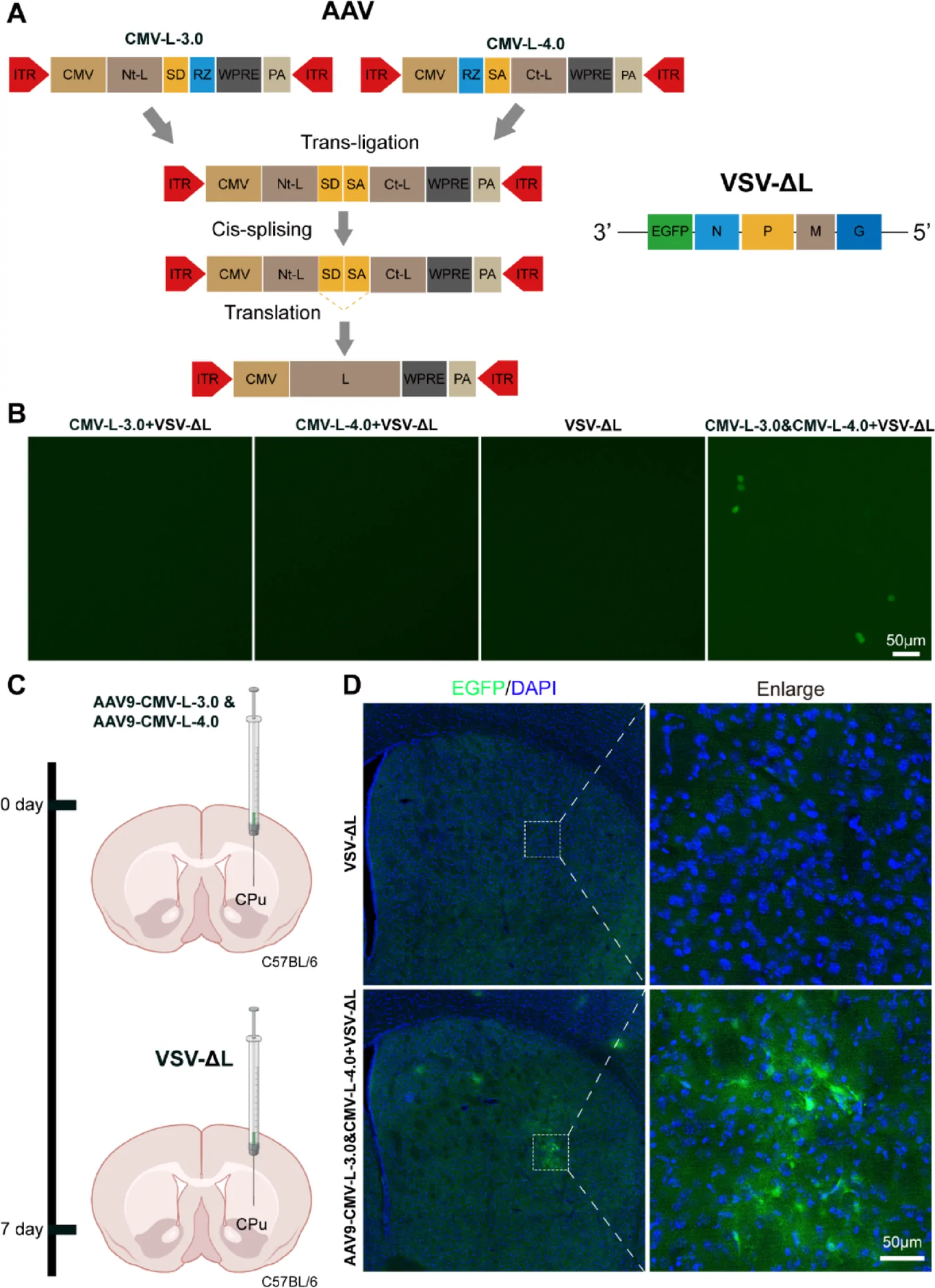
AAV-StitchR system enables large gene delivery in neural cells. **A** Schematic illustration of the constructed AAV-StitchR system for large gene reconstitution. The full-length 6.4 kb VSV-L gene was split into 5′ and 3′ fragments, which were separately assembled into two CMV-driven split expression vectors fused with Twister self-cleaving ribozymes. For brevity, CMV-L-3.0 denotes CMV-Nt-L-SD-RZ-WPRE-PA, and CMV-L-4.0 denotes CMV-RZ-SA-Ct-L-WPRE-PA. The VSV-ΔL viral genome used in this experiment lacks the endogenous L gene. **B** In vitro validation of L gene reconstitution mediated by the StitchR system in HEK-293 T cells. Functional full-length L gene was reconstituted and EGFP expression was activated only in cells co-transfected with both split-L plasmids followed by VSV-ΔL infection. **C** Schematic diagram of viral vector injection in mouse brain. **D** In vivo functional verification of L gene reconstitution via the AAV-StitchR system. The AAV-StitchR could efficiently reconstruct functional L protein to support the transcriptional activity of VSV-ΔL and drive EGFP expression

To verify the feasibility of delivering large genes in neural cells via AAV combined with the seamless StitchR mRNA splicing system, the full-length 6.4 kb vesicular stomatitis virus (VSV) L polymerase (VSV-L) gene was selected for functional validation. VSV is a negative-sense RNA virus whose genome encodes five proteins: N, P, M, G and L. The L gene encodes a multifunctional large polymerase possessing RNA polymerization, mRNA capping, methylation and polyadenylation activities, and it must form a complex with the P protein to exert catalytic functions. Deletion of the L gene from the VSV genome (VSV-ΔL) completely abolishes viral transcription and replication; the reporter gene EGFP carried by VSV-ΔL can only be expressed in the presence of exogenous functional L protein. Given the limited packaging capacity of single AAV vector, the full-length VSV-L coding sequence was split into two fragments to generate two separate expression plasmids, including pAAV-CMV-Nt-L-SD-RZ-WPRE-pA and pAAV-CMV-RZ-SA-Ct-L-WPRE-pA (Fig. 6A). The VSV-ΔL genome harbors N, P, M, G and the EGFP reporter gene (Fig. 6A).

We first performed in vitro functional validation of this system in HEK-293 T cells. Three experimental groups were established: cells transfected with only pAAV-CMV-Nt-L-SD-RZ-WPRE-pA, cells transfected with only pAAV-CMV-RZ-SA-Ct-L-WPRE-pA, and cells co-transfected with the two split-L plasmids. Twenty-four hours after transfection, all groups were infected with an equal dose of VSV-ΔL, alongside a blank control group that received only VSV-ΔL infection. Fluorescence imaging showed that green fluorescent signals were only detectable in the group co-transfected with both split-L plasmids followed by VSV-ΔL infection, indicating that the StitchR system enables intracellular reconstitution of full-length functional VSV-L. Subsequently, the two plasmids were packaged into AAV9 serotype, which exhibits excellent central nervous system tropism. A mixture of the two recombinant AAVs was stereotaxically injected into the CPu region of mouse brains. Seven days after AAV administration, VSV-ΔL was injected into the same brain region, and mice receiving only VSV-ΔL injection served as the in vivo control group (Fig. 6C). Three days after VSV-ΔL injection, mice were perfused transcardially, and brain slices were prepared for fluorescence microscopic imaging. Distinct green fluorescent signals were clearly detected in the CPu of mice co-injected with the dual AAV vectors, whereas no fluorescent signals were observed in the control group injected with VSV-ΔL alone (Fig. 6D).

Collectively, these results demonstrate that the combinatorial strategy of AAV and StitchR-mediated seamless mRNA splicing can achieve efficient delivery and functional reconstitution of large transgenes in neural cells.

## Discussion

In this study, we established an efficient AAV-based gene delivery platform integrating a ribozyme-mediated trans-splicing strategy, cell-type-specific promoters, and high-potency AAV capsids, which enables cell-type-specific expression of genetic cargos in distinct brain cell populations—including local transduction of neurons, astrocytes, and microglia, as well as brain-wide neuronal targeting via systemic administration.

A fundamental limitation of AAV vectors is their restricted packaging capacity (~ 4.7 kb including regulatory elements), which prevents delivery of large or complex genetic sequences such as full-length genes, multi-domain effectors, or polycistronic cassettes. Previous efforts have explored dual-AAV strategies that rely on *trans*-splicing, overlapping, or hybrid recombination to reconstitute large transgenes [27, 28]. However, these methods often suffer from imprecise junctions or low recombination efficiency, leading to truncated or non-functional products. StitchR, a scarless RNA trans-ligation technology, has been developed, in which catalytic ribozymes (small RNA sequences) mediate the reconstitution of a single protein-coding mRNA from two independent AAV vectors. With StitchR, the authors successfully restored large therapeutic muscle proteins—including dysferlin and dystrophin—to endogenous levels, thereby correcting disease pathology in murine models [10]. The StitchR-based dual-AAV delivery strategies established in this study enable efficient transduction of specific neural cell populations, offering a valuable framework for delivering large genes to support functional research on specific cells and the treatment of related diseases.

The successful delivery of genetic material into distinct brain cell types requires not only efficient entry into the target population but also transcriptional precision to avoid off-target expression. In our platform, we systematically matched promoters and AAV capsids to achieve this dual-level specificity. For neuronal targeting, we employed the hSyn promoter, which provides robust and specific expression in neurons across multiple brain regions [29]. Using the natural AAV11 serotype [11], dual-vector co-injection into the striatum resulted in strong local neuronal transduction as well as retrograde labeling of upstream projection neurons, indicating efficient axonal uptake and retrograde transport. This feature is advantageous for circuit-specific genetic labeling and functional manipulation. For astrocyte-specific targeting, the GfaABC1D promoter was utilized in combination with AAV11 [11]. Expression was confined to GFAP-positive astrocytes at the injection site, consistent with prior reports validating GfaABC1D as a compact yet highly specific promoter for astrocytes expression. For microglial targeting, we integrated a microglia-specific mIBA1 promoter and incorporated neuron-detargeting elements (4 × miR-9.T) to minimize ectopic expression. The dual-AAV system achieved efficient, specific expression in IBA1-positive cells, corroborating emerging evidence that AAV11 and related serotypes possess intrinsic microglial tropism when guided by appropriate promoter contexts [12].

While local injections are ideal for spatially restricted studies, broad neuronal delivery across multiple brain regions is essential for systemic therapies and whole-brain functional mapping. Here, we demonstrated that AAVhu.32-PLUS, an engineered capsid variant with high BBB permeability, enabled efficient, neuron-dominant transduction following intravenous co-administration of dual vectors [13]. The success of our dual-vector reconstruction under systemic delivery conditions underscores its translational potential for non-invasive gene therapy.

To achieve more efficient and cell-type-specific delivery of large transgenes, several complementary strategies can be pursued. First, select higher-performance AAV capsids and optimize capsid properties by different means, for example by screening natural serotypes [11, 12, 30], applying rational design to introduce new functionalities [13, 31], using directed evolution to enrich for desired phenotypes [32, 33], or leveraging recent computer-aided design approaches for capsid engineering [34–37]. Second, engineer the vector genome itself, which can include: (1) optimization of regulatory elements such as promoters, enhancers [38], splicing modules and polyadenylation signals; (2) redesign of the transgene cassette (e.g., codon optimization, removal of alternative open reading frames, CpG depletion); and/or (3) protein engineering to enhance payload activity (for example, B-domain–deleted FVIII or the Factor IX Padua variant).

## Materials and methods

### Plasmids construction

First, the core plasmids pAAV-CMV-Nt-EGFP-SD-RZ-WPRE-pA and pAAV-CMV-RZ-SA-Ct-EGFP-WPRE-pA were constructed as backbone vectors for functional validation and subsequent modification. To generate these core plasmids, the Nt-EGFP-SD-RZ and RZ-SA-Ct-EGFP fragments were synthesized (Sangon Biotech (Shanghai) Co., Ltd.) and cloned into pAAV-CMV-EGFP-WPRE-pA (obtained from BrainCase Biotech Co., Ltd.) that had been digested with SalI and EcoRI (Thermo Fisher Scientific, Waltham, MA, USA). The final core constructs, pAAV-CMV-Nt-EGFP-SD-RZ-WPRE-pA and pAAV-CMV-RZ-SA-Ct-EGFP-WPRE-pA, were obtained by homologous recombination. Following the identical cloning strategy, additional plasmids pAAV-CMV-Nt-L-SD-RZ-WPRE-pA and pAAV-CMV-RZ-SA-Ct-L-WPRE-pA were assembled.

The synthesized cell-type–specific promoter sequences hSyn and GfaABC1D were used to replace the CMV promoter in the above core plasmids at the MluI and SalI restriction sites, yielding pAAV-hSyn-Nt-EGFP-SD-RZ-WPRE-pA, pAAV-hSyn-RZ-SA-Ct-EGFP-WPRE-pA, pAAV-GfaABC1D-Nt-EGFP-SD-RZ-WPRE-pA, and pAAV-GfaABC1D-SA-Ct-EGFP-WPRE-pA. (Thermo Fisher Scientific). To construct the pAAV-mIBA1-Nt-EGFP-SD-RZ-WPRE-4 × miR-9.T-pA and pAAV-mIBA1-RZ-SA-Ct-EGFP-WPRE-4 × miR-9.T-pA, the Nt-EGFP-SD-RZ and RZ-SA-Ct-EGFP were cloned into pAAV-mIBA1-EGFP-WPRE-4 × miR-9.T-pA (previously constructed in our lab) that had been digested with SalI and HindIII (Thermo Fisher Scientific, Waltham, MA, USA), producing the two intended mIBA1-driven constructs. Finally, the packaging-plasmid constructions for AAV11 and AAVhu.32-PLUS were performed following previously reported methods.

### Cell culture and AAV vector manufacture

HEK-293 T cells (American Type Culture Collection, Manassas, VA, USA) were maintained in suspension culture using Balance CD medium (Chuangling Cell-wise, Shanghai, China, CW01001) supplemented with 1% penicillin–streptomycin (BasalMedia, Shanghai, China, S110JV) at 37 °C in a humidified incubator with 5% CO₂. Sixteen hours prior to transfection, the cells were transferred to DMEM (BasalMedia, L110KJ) containing 2% fetal bovine serum (Thermo Fisher Scientific GIBCO, Waltham, MA, USA, 10099141C) and 1% penicillin–streptomycin (BasalMedia, S110JV).

Recombinant AAV vectors were produced via transient transfection using a three-plasmid system and linear polyethylenimine (Polysciences, Warrington, PA, USA, 24,765-1). Seventy-two hours post-transfection, viral particles were harvested and purified by iodixanol gradient ultracentrifugation. The purified viruses were then concentrated and buffer-exchanged into PBS containing 0.001% Pluronic F68 (Thermo Fisher Scientific, Waltham, MA, USA, 24,040,032) using Amicon® Ultra centrifugal filters (Merck Millipore, Billerica, MA, USA, UFC910024). Viral genome titers were quantified by qPCR using the iQ SYBR Green Supermix kit (Bio-Rad, Hercules, CA, USA, 1,708,884) with primers WPRE-F (5′-ATGCCTTTGTATCATGCTATTGCT-3′) and WPRE-R (5′-CACGGAATTGTCAGTGCCCAA-3′). The final AAV preparations were aliquoted and stored at − 80 °C until use. The VSV-ΔL was produced by BrainCase Biotechnology Co., Ltd. (Shenzhen, China).

### Research animals

Adult male C57BL/6 mice (8–10 weeks old) were obtained from Hunan SJA Laboratory Animal Company. The mice were housed in a controlled environment under a 12/12-h light/dark cycle within specific pathogen-free facilities. Temperatures were precisely maintained between 22 °C and 24 °C, and humidity levels were kept between 40 and 60%. Both water and food were provided to the animals ad libitum.

### Intravenous and stereotaxic injections

As for intravenous injection, mice were deeply anaesthetized using 1% pentobarbital intraperitoneally (i.p., 50 mg/kg body weight), then fixed on the mouse tail vein syringe. The tail vascularity of mice can be inflated with a 75% alcohol cotton ball, and then injected with viruses using a 1 ml insulin needle, 3 weeks after injection, mice were euthanized and tissue were harvested. As for stereotaxic injection, mice were deeply anesthetized with an intraperitoneal injection of 1% pentobarbital sodium (50 mg/kg body weight) and secured in a stereotaxic frame (RWD Life Science, China; models 68,025 and 68,030). Stereotaxic coordinates were determined based on The Mouse Brain in Stereotaxic Coordinates, 4th Edition by Paxinos and Franklin [39]. Viral suspensions were delivered into specific brain regions, including the CPu (AP + 0.80 mm, ML ± 2.00 mm, DV − 3.30 mm) relative to bregma. Injections were performed at a constant rate of 0.03 μL/min using a stereotaxic injector connected to a fine glass micropipette (Stoelting, Wood Dale, IL, USA; 53,311). After injection, the pipette was left in place for 10 min before being slowly withdrawn. Mice were then placed on a heating pad to recover from anesthesia. Three weeks post-injection, animals were euthanized, and brains were perfused transcardially with PBS followed by 4% paraformaldehyde for tissue collection.

### Immunofluorescence and imaging

Brain slices preparation and imaging were performed according to a previously reported method [40]. The brains were immersed in a 4% paraformaldehyde solution overnight, then dehydrated in a 30% sucrose solution. Subsequently, they were sectioned into 40 μm-thick slices using a microtome (Thermo Fisher Scientific, Waltham, MA, USA), collected in antifreeze solution, and stored at -20 °C until further use. For NeuN staining, sections were incubated with the rabbit anti-NeuN (1:800, Abcam, Cambridge, MA, USA) and then with Goat Anti-Rabbit IgG H&L Alexa Fluor 647 (1:400, The Jackson Laboratory, Bar Harbor, ME, USA, 111–605-003) secondary antibody. For GFAP staining, sections were incubated with the primary antibody goat anti-GFAP (1:800, Abcam, Cambridge, MA, USA, ab53554), followed by the secondary antibody rabbit anti-goat IgG conjugated with Cy3 (1:400, The Jackson Laboratory, Bar Harbor, ME, USA, 305–165-003).For IBA1 staining, sections were incubated with a primary antibody rabbit anti-IBA1 (1:600, Abcam, Cambridge, MA, USA, ab178846), followed by a secondary antibody donkey anti-rabbit IgG conjugated with Cy3 (1:400, The Jackson Laboratory, Bar Harbor, ME, USA, 711-165-152). After PBS washing, all brain slices mounted on microscope slides were counterstained with DAPI (1:4000, Beyotime, Shanghai, China) and sealed with 70% glycerol. Imaging was conducted using either the Olympus VS120 Slide Scanner microscope (Olympus, Tokyo, Japan).

### Data analysis

Data were analyzed using GraphPad Prism 9.0 (GraphPad Software, La Jolla, CA, USA), quantitative statistics were completed using ImageJ v1.8.0 (National Institutes of Health, Bethesda, MD, USA). To quantify the specificity of viral infection, a region of interest (ROI) with a fixed area centered on the injection site was defined, and the number of fluorescent signals within the corresponding ROI was counted across three representative brain slices. Data were presented as means ± standard error of the mean (SEM).

## Supplementary Information

Below is the link to the electronic supplementary material.

Supplementary Material 1

## Data Availability

All datasets for this study are included in the manuscript.
