# Supplementary material for: StitchR-based dual-AAV delivery strategies enable efficient transduction of defined neural cell populations
Source: Mol Brain. 2026 Jul 24;19:74. doi: 10.1186/s13041-026-01331-0 (PMC13685140; doi:10.1186/s13041-026-01331-0)
Supplement: Supplementary file 1 [file 13041_2026_1331_MOESM1_ESM.docx]

**StitchR-based dual-AAV delivery strategies enable efficient transduction of defined neural cell populations**

Nengsong Luo^1,2,3,#^, Yunling Gao^4,#^, Zengpeng Han^1^, Peng Su^6^, Fuqiang Xu^1,2,3,5,^*, Kunzhang Lin^1,5,^*

*^1^ Shenzhen Key Laboratory of Viral Vectors for Biomedicine, Shenzhen-Hong Kong Institute of Brain Science, Shenzhen Institutes of Advanced Technology, Chinese Academy of Sciences, 518055 Shenzhen, P. R. China.*

*^2^ Wuhan National Laboratory for Optoelectronics, Huazhong University of Science and Technology, 430074 Wuhan, P. R. China.*

*^3^ Key Laboratory of Magnetic Resonance in Biological Systems, State Key Laboratory of Magnetic Resonance and Atomic and Molecular Physics, National Center for Magnetic Resonance in Wuhan, Innovation Academy for Precision Measurement Science and Technology, Chinese Academy of Sciences, 430071 Wuhan, P. R. China.*

*^4^ Institute of Neuroscience and Brain Diseases, Xiangyang Central Hospital, Affiliated Hospital of Hubei University of Arts and Science, 441106 Xiangyang, P. R. China.*

*^5^ Key Laboratory of Quality Control Technology for Virus-Based Therapeutics, Guangdong Provincial Medical Products Administration, NMPA Key Laboratory for Research and Evaluation of Viral Vector Technology in Cell and Gene Therapy Medicinal Products, the Brain Cognition and Brain Disease Institute, Shenzhen Institutes of Advanced Technology, Chinese Academy of Sciences, 518055 Shenzhen, P. R. China.*

*^6^ Brain Case Biotechnology Co., Ltd., 518107 Shenzhen, P. R. China.*

** Correspondence:* *kz.lin@siat.ac.cn (K.L.); fq.xu@siat.ac.cn (F.X.)*

*# These authors contributed equally to this work.*

pITR-CMV-Nt-EGFP-SD-RZ-WPRE-pA:

ctgcgcgctcgctcgctcactgaggccgcccgggcaaagcccgggcgtcgggcgacctttggtcgcccggcctcagtgagcgagcgagcgcgcagagagggagtggccaactccatcactaggggttcctgcggccgcacgcgtgacattgattattgactagttattaatagtaatcaattacggggtcattagttcatagcccatatatggagttccgcgttacataacttacggtaaatggcccgcctggctgaccgcccaacgacccccgcccattgacgtcaataatgacgtatgttcccatagtaacgccaatagggactttccattgacgtcaatgggtggagtatttacggtaaactgcccacttggcagtacatcaagtgtatcatatgccaagtacgccccctattgacgtcaatgacggtaaatggcccgcctggcattatgcccagtacatgaccttatgggactttcctacttggcagtacatctacgtattagtcatcgctattaccatggtgatgcggttttggcagtacatcaatgggcgtggatagcggtttgactcacggggatttccaagtctccaccccattgacgtcaatgggagtttgttttggcaccaaaatcaacgggactttccaaaatgtcgtaacaactccgccccattgacgcaaatgggcggtaggcgtgtacggtgggaggtctatataagcagagctggatcctctagagtcgacgccaccatggtgagcaagggcgaggagctgttcaccggggtggtgcccatcctggtcgagctggacggcgacgtaaacggccacaagttcagcgtgtccggcgagggcgagggcgatgccacctacggcaagctgaccctgaagttcatctgcaccaccggcaagctgcccgtgccctggcccaccctcgtgaccaccctgacctacggcgtgcagtgcttcagccgctaccccgaccacatgaagcagcacgacttcttcaagtccgccatgcccgaaggtaagtatcaaggttacaagacaggtttaaggagaccaatagaaactgggctaacactgccaatgccggtcccaagcccggataaaagtggaggggcccagaattcgatatcaagcttatcgataatcaacctctggattacaaaatttgtgaaagattgactggtattcttaactatgttgctccttttacgctatgtggatacgctgctttaatgcctttgtatcatgctattgcttcccgtatggctttcattttctcctccttgtataaatcctggttgctgtctctttatgaggagttgtggcccgttgtcaggcaacgtggcgtggtgtgcactgtgtttgctgacgcaacccccactggttggggcattgccaccacctgtcagctcctttccgggactttcgctttccccctccctattgccacggcggaactcatcgccgcctgccttgcccgctgctggacaggggctcggctgttgggcactgacaattccgtggtgttgtcggggaaatcatcgtcctttccttggctgctcgcctgtgttgccacctggattctgcgcgggacgtccttctgctacgtcccttcggccctcaatccagcggaccttccttcccgcggcctgctgccggctctgcggcctcttccgcgtcttcgccttcgccctcagacgagtcggatctccctttgggccgcctccccgcatcgataccgagcgctgctcgagagatctacgggtggcatccctgtgacccctccccagtgcctctcctggccctggaagttgccactccagtgcccaccagccttgtcctaataaaattaagttgcatcattttgtctgactaggtgtccttctataatattatggggtggaggggggtggtatggagcaaggggcaagttgggaagacaacctgtagggcctgcggggtctattgggaaccaagctggagtgcagtggcacaatcttggctcactgcaatctccgcctcctgggttcaagcgattctcctgcctcagcctcccgagttgttgggattccaggcatgcatgaccaggctcagctaatttttgtttttttggtagagacggggtttcaccatattggccaggctggtctccaactcctaatctcaggtgatctacccaccttggcctcccaaattgctgggattacaggcgtgaaccactgctcccttccctgtcctt

pITR-CMV-RZ-SA-Ct-EGFP-WPRE-pA:

ctgcgcgctcgctcgctcactgaggccgcccgggcaaagcccgggcgtcgggcgacctttggtcgcccggcctcagtgagcgagcgagcgcgcagagagggagtggccaactccatcactaggggttcctgcggccgcacgcgtgacattgattattgactagttattaatagtaatcaattacggggtcattagttcatagcccatatatggagttccgcgttacataacttacggtaaatggcccgcctggctgaccgcccaacgacccccgcccattgacgtcaataatgacgtatgttcccatagtaacgccaatagggactttccattgacgtcaatgggtggagtatttacggtaaactgcccacttggcagtacatcaagtgtatcatatgccaagtacgccccctattgacgtcaatgacggtaaatggcccgcctggcattatgcccagtacatgaccttatgggactttcctacttggcagtacatctacgtattagtcatcgctattaccatggtgatgcggttttggcagtacatcaatgggcgtggatagcggtttgactcacggggatttccaagtctccaccccattgacgtcaatgggagtttgttttggcaccaaaatcaacgggactttccaaaatgtcgtaacaactccgccccattgacgcaaatgggcggtaggcgtgtacggtgggaggtctatataagcagagctggatcctctagagtcgacagccttaacactgccaatgccggtcccaagcccggataaaagtggagggaggcttgtcgagacagagaagactcttgcgtttctgataggcacctattggtcttactgacatccactttgcctttctctccacaggctacgtccaggagcgcaccatcttcttcaaggacgacggcaactacaagacccgcgccgaggtgaagttcgagggcgacaccctggtgaaccgcatcgagctgaagggcatcgacttcaaggaggacggcaacatcctggggcacaagctggagtacaactacaacagccacaacgtctatatcatggccgacaagcagaagaacggcatcaaggtgaacttcaagatccgccacaacatcgaggacggcagcgtgcagctcgccgaccactaccagcagaacacccccatcggcgacggccccgtgctgctgcccgacaaccactacctgagcacccagtccgccctgagcaaagaccccaacgagaagcgcgatcacatggtcctgctggagttcgtgaccgccgccgggatcactctcggcatggacgagctgtacaagtaagaattcgatatcaagcttatcgataatcaacctctggattacaaaatttgtgaaagattgactggtattcttaactatgttgctccttttacgctatgtggatacgctgctttaatgcctttgtatcatgctattgcttcccgtatggctttcattttctcctccttgtataaatcctggttgctgtctctttatgaggagttgtggcccgttgtcaggcaacgtggcgtggtgtgcactgtgtttgctgacgcaacccccactggttggggcattgccaccacctgtcagctcctttccgggactttcgctttccccctccctattgccacggcggaactcatcgccgcctgccttgcccgctgctggacaggggctcggctgttgggcactgacaattccgtggtgttgtcggggaaatcatcgtcctttccttggctgctcgcctgtgttgccacctggattctgcgcgggacgtccttctgctacgtcccttcggccctcaatccagcggaccttccttcccgcggcctgctgccggctctgcggcctcttccgcgtcttcgccttcgccctcagacgagtcggatctccctttgggccgcctccccgcatcgataccgagcgctgctcgagagatctacgggtggcatccctgtgacccctccccagtgcctctcctggccctggaagttgccactccagtgcccaccagccttgtcctaataaaattaagttgcatcattttgtctgactaggtgtccttctataatattatggggtggaggggggtggtatggagcaaggggcaagttgggaagacaacctgtagggcctgcggggtctattgggaaccaagctggagtgcagtggcacaatcttggctcactgcaatctccgcctcctgggttcaagcgattctcctgcctcagcctcccgagttgttgggattccaggcatgcatgaccaggctcagctaatttttgtttttttggtagagacggggtttcaccatattggccaggctggtctccaactcctaatctcaggtgatctacccaccttggcctcccaaattgctgggattacaggcgtgaaccactgctcccttccctgtcctt
